# The Amazing World of IDPs in Human Diseases

**DOI:** 10.3390/biom11020333

**Published:** 2021-02-23

**Authors:** Simona Maria Monti, Giuseppina De Simone, Emma Langella

**Affiliations:** Institute of Biostructures and Bioimaging, CNR, via Mezzocannone 16, I-80134 Naples, Italy; gdesimon@unina.it (G.D.S.); emma.langella@cnr.it (E.L.)

It has been clearly established that some proteins or protein regions are devoid of any stable secondary and/or tertiary structure under physiological conditions, but still possess fundamental biological functions [1]. These intrinsically disordered proteins (IDPs) or regions (IDRs) have peculiar features due to their plasticity, such as the capacity for binding their biological targets with high specificity and low affinity, and the possibility of interaction with numerous partners [2,3]. IDPs and IDRs are especially prevalent in eukaryotes, suggesting that disorder in nucleated cells is associated with many key functions, such as signaling and regulation. However, a correlation between intrinsic disorder and various human diseases such as cancer, diabetes, amyloidosis, and neurodegenerative diseases is also evident, highlighting the importance of this topic [4,5]. For the present Special Issue, we have invited researchers to contribute with original research articles, as well as reviews, on the amazing world of the IDPs or IDRs involved with human diseases. We have brought together an internationally recognized team of researchers who work in this field. The contributing authors have presented important and novel aspects of disorder, either correlated with specific medical diseases, or aimed to increase the basic understanding of intrinsic disorder. 

The work presented by Tsvetkov and colleagues investigated the degradation of IDPs via a unique class of 26S proteasome that is free of ATP [6]. The authors found that NADH-stabilized 26S proteasome complexes promote the efficient degradation of many IDPs that might not require ATP-dependent unfolding, such as p27, Tau, c-Fos, and more. This interesting finding exemplifies a new principle of how mitochondria, with a key role in NADH production, might be involved in IDP/IDR homeostasis.

Ortega-Alarcon and co-workers presented an investigation on MeCP2 [7], which is an intrinsically disordered multi-domain protein and a potential pharmacological target associated with Rett syndrome (RTT). The authors report an in-depth biophysical study of two mutant variants of MeCP2 associated with RTT using different protein constructions in order to evaluate the effect of the protein-disordered regions on structural stability, conformation, and DNA binding ability. The results obtained lead to a more general reflection on the molecular context-dependent effects induced by mutations in proteins.

The interaction between the intrinsically disordered NUPR1 and the human importinα3 (Impα3) was investigated in depth by Neira et al. [8] using several spectroscopic and biophysical techniques, including NMR and molecular docking. The authors focused on the affinity of the Nuclear Localization Sequence (NLS) of NUPR1 towards Impα3, taking into account several mutants of the NLS region, and demonstrated that the phosphorylation of Thr68 induces a conformational switch in the NLS region of NUPR1 which hampers binding to Impα3. From a more general point of view, the study allows the detection of key residues able to modulate NUPR1 interactions with its partners.

Pajkos and colleagues carried out an interesting in silico analysis concerning the evolutionary origin of intrinsically disordered regions that are specifically targeted in cancer [9]. The authors focused on a subset of cancer genes belonging to the class of IDPs and used a novel conservation and phylogenetic-based strategy. By means of several case studies, the authors conclude that the disordered cancer risk regions showed remarkable conservation with ancient evolutionary origin, highlighting their importance in biological processes.

The paper by Wong et al. analyzes the enrichment patterns of missense mutation-causing single nucleotide variants (SNVs) that are associated with disease and cancer, as well as those present in the healthy population, in protein–protein complexes showing IDR-mediated interactions [10]. Notably, data analysis indicates a strong enrichment at the interface core of interacting IDRs in disease mutations and its depletion in neutral ones, thus supporting the disruption of IDR interactions as a common mechanism for many diseases. In conclusion, the authors highlight the importance of understanding and predicting the effect of missense mutations on disease susceptibility.

The investigation of *Helicobacter pylori* UreG structural dynamics in solution was carried out by Pierro and colleagues [11], who reported the effects of physiological cofactors Ni(II) and GTP on protein mobility by using techniques such as isothermal titration calorimetry and site-directed spin labeling coupled to electron paramagnetic spectroscopy. The obtained results, which showed that the concomitant addition of both Nickel(II) and GTP induces a modification of the structure and mobility in two regions of the protein, may provide perspectives for future research on molecules with anti-bacterial activities to overcome anti-microbial resistance (AMR).

An interesting study was conducted by Gadhave and colleagues on the disorder content of the ubiquitin proteasome system (UPS) [12], which plays a key role in the pathogenesis of various types of cancers and neurodegenerative diseases. By means of five different IDP prediction tools, authors classified the disease-associated UPS proteins in highly ordered, moderately disordered, and highly disordered proteins. Concurrently, multiple post-translational modification sites were identified, mainly located in the disordered regions of proteins. Since these proteins interact with their biological partners for the normal functioning of protein homeostasis, a complete elucidation of the roles of the identified IDPRs and disorder-based binding regions in the pathogenesis of diseases is of great importance for biomedical research. 

Finally, two reviews enriched our Special Issue with a wide and extensive discussion on certain topics [13,14]. The review by Sokolik and co-workers gave a structural overview on the fascinating WASp-interacting protein (WIP), which is a regulator of actin cytoskeleton assembly and remodeling, a cellular multi-tasker, and a key member of a network of protein–protein interactions [13]. The authors provided a deeper understanding of the mechanisms by which WIP mediates its biological functions, which have an impact on health and disease, paving the way for a better understanding of key biological processes with potential therapeutic implications. Last, but not least, Kim and coworkers overviewed the features revealed by NMR spectroscopy of monomeric alpha-synuclein, for which the aggregation is strongly correlated with Parkinson’s disease [14].

In conclusion, in this Special Issue, examples of recent progress in the IDPs/IDRs have been reported with the aim to contribute to advancing the field of the intrinsic disorder issue in human disease and encourage further research on the topic.
